# Role of N6-methyl-adenosine modification in mammalian embryonic development

**DOI:** 10.1590/1678-4685-GMB-2020-0253

**Published:** 2021-05-14

**Authors:** Chengshun Li, Ziping Jiang, Jindong Hao, Da Liu, Haobo Hu, Yan Gao, Dongxu Wang

**Affiliations:** 1 Jilin University, College of Animal Science, Laboratory Animal Center, Changchun, China.; 2 The First Hospital of Jilin University, Department of hand surgery, Changchun, China.; 3 Changchun University of Chinese Medicine, Department of Pharmacy, Changchun, China.

**Keywords:** m6A modification, embryo development, expression pattern, epigenetic reprogramming

## Abstract

N6-methyl-adenosine (m6A) methylation is one of the most common and abundant modifications of RNA molecules in eukaryotes. Although various biological roles of m6A methylation have been elucidated, its role in embryonic development is still unclear. In this review, we focused on the function and expression patterns of m6A-related genes in mammalian embryonic development and the role of m6A modification in the embryonic epigenetic reprogramming process. The modification of m6A is regulated by the combined activities of methyltransferases, demethylases, and m6A-binding proteins. m6A-related genes act synergistically to form a dynamic, reversible m6A pattern, which exists in several physiological processes in various stages of embryonic development. The lack of one of these enzymes affects embryonic m6A levels, leading to abnormal embryonic development and even death. Moreover, m6A is a positive regulator of reprogramming to pluripotency and can affect embryo reprogramming by affecting activation of the maternal-to-zygotic transition. In conclusion, m6A is involved in the regulation of gene expression during embryonic development and the metabolic processes of RNA and plays an important role in the epigenetic modification of embryos.

## Introduction

Modifications have been identified in different types of RNA, including mRNAs, tRNA, rRNA, and snoRNA, which are associated with various biological functions in mammals (Machnicka et al., 2013). RNA methylation has an important role in mRNA modifications, such as N6-methyl-adenosine (m6A) modification, which is the most common methylation modification (Niu et al., 2013; Frye et al., 2018). m6A was found to be highly enriched in 3’ untranslated regions (UTR) or long internal exons, and 5’ UTRs (Dominissini et al., 2012; Meyer et al., 2012; Batista et al., 2014; Ke et al., 2015; Zhang and Hamada, 2018). Moreover, m6A modifications are ubiquitous in prokaryotes, yeasts, and viruses (Chandola et al., 2015). Indeed, m6A is crucial for long non-coding RNAs, small nuclear RNAs, and ribosomal RNAs (Shimba et al., 1995; Piekna-Przybylska et al., 2008; Patil et al., 2016).

Most previous studies focused on gene and protein expression via DNA modification, such as DNA methylation and histone modifications (Toh et al., 2017; Xiang et al., 2019). However, there is limited research on RNA methylation modifications during embryonic development. RNA is widely involved in mammalian reproduction processes, such as the maturation of sperm and oocytes, embryonic development, and maintenance of the pluripotency of embryonic stem cells (Bettegowda and Smith, 2007; Sha et al., 2020). Previous reports showed that RNA was essential for embryonic and fetal development (Giraldez et al., 2005; Murchison et al., 2005; Melton *et al*., 2010; Stadler et al., 2010; Suh et al., 2010).

In this review, we analyzed the function and expression patterns of m6A-related genes in mammalian embryonic development. In addition, the role of m6A modification in the embryonic epigenetic reprogramming process was summarized.

## Effect of m6A methylation-related genes on embryonic development

### Writers

**Figure 1 -:**
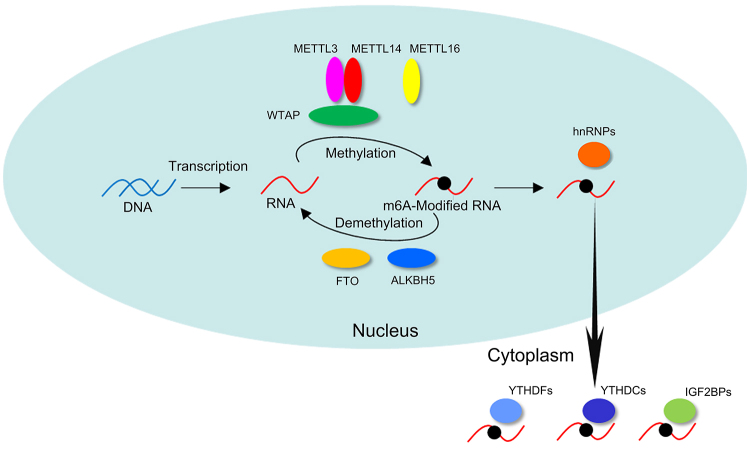
The m6A modification process. METTL3, METTL14, WTAP, and METTL16 are methyltransferases, termed as “writers,” involved in m6A modification in the nucleus. FTO and ALKBH5 are demethylases, termed as “erasers.” YTHDFs, YTHDCs, and IGF2BPs are methylated reader proteins, referred to as “readers,” located in the cytoplasm. Writers mediate m6A methylation, which is a post-transcriptional process in RNA. Erasers catalyze the demethylation of m6A, which reverses methylation. Readers recognize m6A modifications, which play a role in mRNA decay and translation.

m6A modification is regulated by several proteins including methyltransferase-like 3 (METTL3), methyltransferase-like 14 (METTL14), WTAP, and methyltransferase-like 16 (METTL16) referred to as “writers.” They catalyze the m6A methylation of mRNA bases (Figure 1). METTL3 methylates the sequence motif RRACH (R = A or G; H = A, C or U) (Mendel et al., 2018). The heterodimer complex METTL3/METTL14 catalyzes the m6A methylation modification of RNA in mammalian cells (Bokar et al., 1997; Wang Y et al., 2014; Meng et al., 2019). WTAP was the third protein identified in the m6A methyltransferase complex (Ping et al., 2014). Although WTAP has no methylation activity, it recruits METTL3/METTL14 heterodimer and promotes m6A methylation (Liu J et al., 2014). Previous studies reported that WTAP knockdown led to embryonic defects in mammals (Ping *et al*., 2014). The knockdown of METTL3 reduced m6A levels during mammalian embryonic development (Geula et al., 2015). The loss of METTL3 expression led to decreased m6A levels, which caused embryonic cells to remain in the naive state and consequently, resulted in embryonic lethality during the implantation phase (Table 1). METTL3 expression is important in normal meiosis, and the loss of expression of METTL3 led to defects in sperm meiosis (Geula *et al*., 2015; Xu et al., 2017). Indeed, METTL3 is required for development of the cerebellum , neurogenesis, and skin in mammals (Yoon et al., 2017; Wang C *et al*., 2018; Xi et al., 2020). In addition, METTL14 is indispensable for mouse post-implantation development (Meng *et al*., 2019). METTL16 catalyzes the methylation of the specific sequence UACAGAGAA in structured RNAs (Pendleton et al., 2017). In a previous study, the knockout of METTL16 led to the dysregulation of gene expression, which in turn, resulted in the failure of further development of implanted embryos (Mendel *et al*., 2018). These findings suggested that methyltransferase-mediated m6A modification is necessary for embryonic development.

**Table 1 -:** m^6^A methylation related to embryo development.

m^6^A-related protein	Function	Position	Knockout effect	References
Methyltransferase (Writers)				
METTL3	RNA m^6^A methylation	Nucleus	Embryo death	(Geula *et al*., 2015)
			Stagnation of sperm meiosis	(Xu *et al*., 2017)
METTL14	RNA m^6^A methylation	Nucleus	Embryo death	(Meng *et al*., 2019)
			Stagnation of sperm meiosis	(Lin *et al*., 2017)
METTL16	RNA m^6^A methylation	Nucleus	Developmental arrest when implantation	(Mendel *et al*., 2018)
WTAP	Regulatory subunit in m^6^A methylation	Nucleus	Embryonic developmental defect	(Ping *et al*., 2014)
Demethylase (Erasers)				
FTO	Demethylation of m^6^A RNA	Nucleus	Slow fetal growth	(Osborn *et al*., 2014)
ALKBH5	Demethylation of m^6^A RNA	Nucleus	Sperm apoptosis	(Zheng *et al*., 2013)
Binding proteins (Readers)				
YTHDC1	Identify m6A RNA	Nucleus	Embryo death	(Kasowitz *et al*., 2018)
			Gametogenesis arrest	(Hsu *et al*., 2017)
YTHDC	Identify m6A RNA	Nucleus	Adult fertility loss	(Zhao *et al*., 2017)
YTHDF2	Identify m6A RNA	Cytoplasm	Oocyte developmental defect	(Ivanova *et al*., 2017)
IGF2BP1	Identify m6A RNA	Cytoplasm	Embryo developmental defect	(Hao *et al*., 2020)

### Erasers

The demethylases involved in m6A modification are collectively known as “erasers” and include FTO and ALKBH5, belonging to the ALKB family (Kurowski et al., 2003; Gerken et al., 2007; Fu et al., 2010). FTO and ALKBH5 have been demonstrated to play a role in the reversible methylation of m6A *in vitro* and *in vivo* (Jia et al., 2011). FTO has been found to play a role in obesity and energy metabolism (Frayling et al., 2007; Fischer et al., 2009; Church et al., 2010; Yang et al., 2012). A recent study suggested that FTO is a demethylase involved in m6A modification (Jia *et al*., 2011). Reduction in the expression of FTO, which was detected in the nucleus and colocalized with nuclear speckles, induced weight loss or early death in mice (Fischer *et al*., 2009; Osborn et al., 2014). An increase in FTO expression levels, caused by GSK-3 deletion or inhibition, induced a decrease in m6A modification levels in mouse embryonic stem cells and reduced pluripotency (Faulds et al., 2018). ALKBH5 is the second mammalian m6A demethylase and ALKBH5-dependent m6A demethylation affects mRNA export (Zheng et al., 2013). Furthermore, ALKBH5 regulates spermatogenesis by reversing m6A methylation modification, resulting in the apoptosis of spermatocytes and impaired fertility in ALKBH5-deficient male mice. Based on these findings, it can be suggested that demethylases are involved in the dynamic process of m6A modification, which plays important roles in RNA export and metabolism, RNA processing factor assembly, and gene expression in mammalian embryonic development.

### Readers

Methylated reading proteins, called “readers,” are required to bind to m6A and perform the downstream functions of m6A modification. Readers include YTH domain proteins (YTHDF1, YTHDF2, YTHDF3, YTHDC1 and YTHDC2) and insulin-like growth factor 2 mRNA-binding proteins (IGF2BPs; including IGF2BP1, IGF2BP2 and IGF2BP3). YTHDF1, YTHDF2, and YTHDF3 are cytoplasmic readers. YTHDF1 and YTHDF3 work in concert to affect the translation of m6A RNA (Wang X et al., 2015; Shi et al., 2017) while YTHDF2 expedites mRNA decay (Wang X *et al*., 2014). The loss of YTHDF2 expression led to an imbalance in gene transcript regulation and affected oocyte development in mice (IvaZnova et al., 2017). These findings indicated that YTHDF2 was a crucial factor in the acquisition of oocyte competence and during early zygotic development. YTHDC1 is essential for embryonic development in mice and localizes to the nucleus (Kasowitz et al., 2018). The inactivation of YTHDC1 leads to embryonic lethality. A previous study showed that although YTHDC2 knockout mice reached adulthood, both male and female mice were infertile (Hsu et al., 2017b). When readers bind to m6A-modified RNA, the targets of m6A-modified transcripts are activated during embryonic development. The loss of YTHDC2 expression caused abnormal m6A modification in mice (Wojtas et al., 2017). Thus, YTH domain proteins maintain mRNA stability and translation through m6A modification during embryonic development. IGF2BPs and YTHDF2 have a different function in the recognition and regulation of m6A RNA (Huang et al., 2018). Compared to YTH domain proteins, which mediate the mRNA decay pathway, IGF2BPs guard m6A-modified mRNA from decay. IGF2BPs, such as IGF2BP1, play roles in inhibiting mRNA degradation, increasing mRNA stability, and facilitating translation (Hao et al., 2020). Moreover, IGF2BPs as RNA-binding protein, play a potentially key role in embryonic development (Degrauwe et al., 2016). These data indicated that IGF2BPs are involved in various RNA biological processes in embryos at different developmental stages.

## Expression pattern of the genes responsible for regulating the m6A modification

Increasing data have demonstrated that RNA modification is a dynamic process involved in the regulation of a variety of physiological processes (Motorin and Helm, 2011; Chan *et al*., 2012; Meyer and Jaffrey, 2014; Kirchner and Ignatova, 2015). Our previous data confirmed that m6A modification is a dynamic process that occurs during mouse embryonic development (Hao et al., 2019). This finding indicated that the methylation status of m6A was dynamically regulated by m6A-related genes during embryonic development (Faulds et al., 2018). The expression of m6A-related genes, such as *METTL3*, is important for the regulation of m6A modification during different embryonic stages (Kwon et al., 2019). In general, m6A levels increase slowly at the 2-cell, 4-cell, and 8-cell stages of mouse embryos. Notably, the factors associated with cellular pluripotency, such as OCT4, SOX2, and NANOG, were highly expressed in mouse embryonic cells. However, knocked down *METTL3* and *METTL14* reduced the expression of these pluripotency-related genes during embryonic development (Wang Y et al., 2014). m6A modification regulated gene expression, indicating that m6A modification determined RNA fate (i.e., transcription, splicing, or degradation) in the embryonic differentiation process (Young, 2011; Dunn et al., 2014; Yu et al., 2018). Compared to the 2-cell stage, the blastocyst stage exhibited increased m6A levels because of the active transcription of numerous genes. Thus, m6A levels show dynamic changes with increased expression during embryonic development.

**Figure 2 -:**
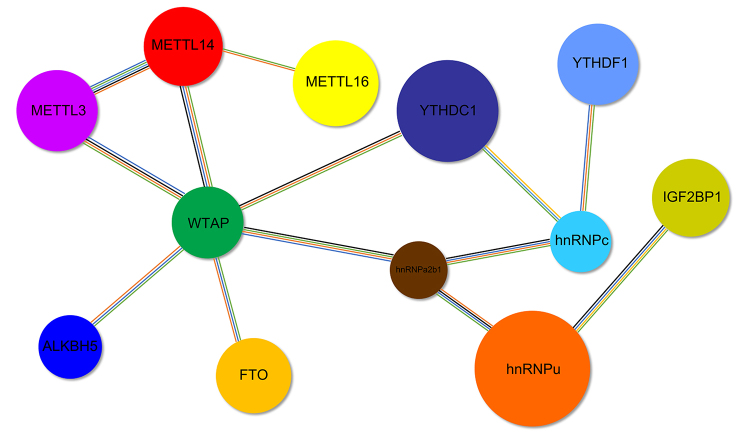
Network of m6A-related genes. WTAP is a regulatory subunit required for the METTL3/METTL14 heterodimer complex. METTL16 is essential for RNA binding and methylation activity, and is associated with METTL14. FTO and ALKBH5 mediate RNA demethylation at the m6A modification site, causing unmethylated RNA to be bound by HuR protein, leading to constitutive expression. YTHDC1, YTHDF1, and IGF2BP1 recognize the methylation site and regulate mRNA degradation. hnRNPs regulate the expression of YTHDC1, YTHDF1, and IGF2BP1 in m6A modification.

The dynamic changes in m6A levels are attributed to methyltransferases (writers), demethylases (erasers), and methylation reader proteins (readers). These m6A-related genes are mainly involved in the production and removal of m6A RNA as well as the stabilization, translation, and degradation processes of m6A RNA. The network of methyltransferases, demethylases, and methylation reader proteins was analyzed using STRING bioinformatics software (Figure 2).METTL3/METTL14 and WTAP as the core were connected to erasers and readers. Heterogeneous nuclear ribonucleoproteins (hnRNPs) such as hnRNP a2/b1 play important roles in m6A modification (Wu et al., 2018). HnRNP family members are mediators of m6A modification, which switch on RNA structural and regulated RNA-protein interactions (Liu N et al., 2015). Moreover, hnRNPu was found to be associated with IGF2BP1, which regulated m6A levels during mouse embryonic development (Weidensdorfer et al., 2009). In addition, hnRNPA2/B1 is crucial for gene transcripts and embryonic stem cell differentiation regulated by METTL3-dependent m6A modification (Kwon et al., 2019). Furthermore, YTHDC1 and YTHDF1 influence mRNA splicing as m6A readers, whereas hnRNPc binds to m6A-modified RNAs to promote mRNA processing and alternative splicing (Hsu et al., 2017a). These data indicate that the loss of the expression of any m6A-related genes could lead to abnormal embryonic development in mammals.

## Role of m6A in epigenetic reprogramming of embryos

**Figure 3 -:**
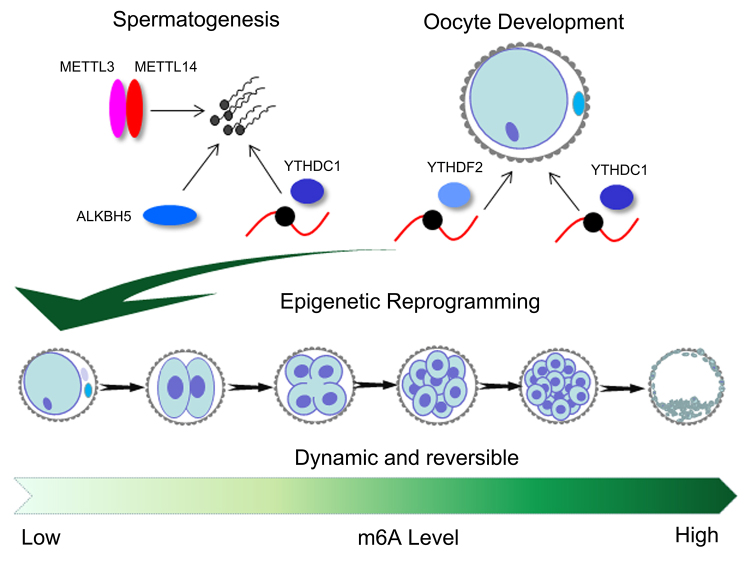
Modification of m6A in embryonic epigenetic reprogramming. METTL3/METTL14, YTHDC1, and ALKBH5 play important roles in spermatogenesis. YTHDC1 and YTHDF2 are crucial in oocyte development. The dynamic and reversible m6A modification processes establish epigenetic reprogramming after fertilization. m6A levels increase during embryonic development.

DNA methylation and histone deacetylation are critical for epigenetic reprogramming during mammalian embryonic development. However, there are limited studies on m6A modification. m6A was found to be enriched in several functional groups, including chordate embryonic development, embryonic development, and gastrulation using m6A-sequencing (seq) analysis (Batista et al., 2014). This finding indicated that, like DNA methylation, dynamic and reversible m6A RNA modification could affect the epigenetic reprogramming of embryos (Figure 3). Increased m6A levels promoted the reprogramming of mouse embryonic fibroblasts into pluripotent stem cells, indicating that m6A modification is a positive regulator of the epigenetic reprogramming of embryos (Chen et al., 2015). The knockout of *METTL3* leads to embryonic death, which might be related to decreases in m6A levels, causing abnormal reprogramming during early embryonic development in mice (Lin et al., 2017).

Maternal-to-zygotic transition (MZT) is a key process in which two differentiated gametes are reprogrammed during embryonic development. The clearance of maternal mRNAs and zygotic genome activation is realized during MZT (Zhao et al., 2017). Many maternal imprinted genes are eliminated through various transcriptional mechanisms to promote genome reprogramming in oocytes (Sui et al., 2020). m6A modification can affect embryonic reprogramming through transcriptional mechanisms involved in maternal clearance, which affect the stability of m6A RNA and promote the degradation of maternal mRNA. YTHDF2 plays an important role in this process by accelerating the degradation of mRNA. Loss of the expression of YTHDF2 did not clear maternal mRNA and induced abnormal embryonic development (Zhao and He, 2017). m6A modification could influence embryonic reprogramming by regulating a variety of cytokines, including cell cycle regulators and pluripotency factors (Chen et al., 2015). Furthermore, m6A modification could promote the translation of key transcriptional activators of zygotes during MZT (Zhao *et al*., 2017). A recent study suggested that m6A modification controlled cell reprogramming to pluripotency (Alarcon et al., 2015). These findings demonstrated that m6A modification regulated epigenetic reprogramming during embryonic development.

m6A modification plays an important role in mouse embryo reprogramming by regulating RNA stability, degradation, translation, alternative splicing, and gene expression. m6A-related genes interact with regulatory factors to ensure the normal epigenetic reprogramming of embryos as a regulatory network.

In conclusion, the dynamic and reversible chemical m6A modification of RNA has shown effects on gene expression regulation and epigenetic reprogramming during embryonic development. The key factors affecting m6A levels are the three types of m6A-related enzymes: methyltransferases, demethylases, and methylation reader proteins. These proteins affect mRNA transcription, splicing, nuclear export, localization, and translation during embryonic development. The loss of expression of these proteins or their decreased activity can lead to the retardation of embryonic development and even death. Moreover, m6A modification plays an important role in the epigenetic reprogramming of embryonic development. Abnormal m6A levels can lead to defects in sperm or oocytes, fertility, and activation of the zygote genome. Thus, abnormal m6A modification leads to abnormal epigenetic reprogramming.
